# Oxaliplatin toxicity presenting as a liver nodule – case report

**DOI:** 10.1186/s12885-015-1247-4

**Published:** 2015-04-10

**Authors:** Henrique Alexandrino, Domingos Oliveira, Maria Augusta Cipriano, Luís Ferreira, J Guilherme Tralhão, Francisco Castro e Sousa

**Affiliations:** 1 Department of General Surgery, Coimbra University Hospital, Coimbra, Portugal; 2 Faculty of Medicine, University of Coimbra, Coimbra, Portugal; 3 Department of Pathology, Oncology Institute, Coimbra, Portugal; 4 Department of Pathology, Coimbra University Hospital, Coimbra, Portugal

## Abstract

**Background:**

Oxaliplatin based chemotherapy is often used as adjuvant therapy in colon and rectal cancer. A reported side effect is Sinusoidal Obstruction Syndrome which is characterized by a spectrum of pathologic changes, from sinusoidal dilation, peri-sinusoidal haemorrhage, peliosis and nodular regenerative hyperplasia. Very rarely it can cause the development of liver nodules mimicking liver metastases. Herein, we report a case of Sinusoidal Obstruction Syndrome causing a liver nodule suspicious of liver metastasis on imaging. This is the third reported case of this complication of oxaliplatin toxicity, in which resection was performed and pathological diagnosis confirmed.

**Case presentation:**

We report the case of a 59 year old man with stage III colon cancer who underwent sigmoidectomy followed by adjuvant chemotherapy with oxaliplatin. One year after surgery a liver nodule was detected and the patient underwent right hepatectomy. Pathology showed no liver nodule and diagnosed sinusoidal obstruction syndrome.

**Conclusion:**

We describe the third reported case of a liver lesion mimicking a liver metastasis after oxaliplatin-based chemotherapy for colon cancer. We suggest that in patients heavily treated with oxaliplatin with *de novo* liver nodules, this differential diagnosis should be considered. In particular, in this population of patients an intense imagiologic evaluation and even a preoperative biopsy should be pursued to confirm the diagnosis of malignancy and avoid overtreatment.

## Background

Oxaliplatin based chemotherapy is often used as adjuvant therapy in colon and rectal cancer or in perioperative therapy in liver metastatic disease [1,2]. A reported side effect is Sinusoidal Obstruction Syndrome (SOS), formerly known as veno-occlusive disease, which is characterized by a spectrum of pathologic changes, from sinusoidal dilation, peri-sinusoidal haemorrhage, peliosis and nodular regenerative hyperplasia [3]. SOS can cause increased morbidity after liver resection [4] but a recently reported side effect is the development of liver nodules mimicking liver metastases [5,6].

Herein, we report a case of SOS causing a liver nodule on imaging suspicious of liver metastasis. This is the third reported case of this complication of oxaliplatin toxicity, in which resection was performed and pathological diagnosis confirmed.

## Case presentation

**Figure 1:**
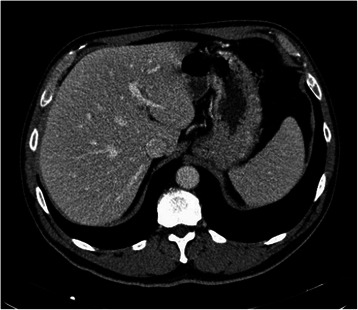
**Contrast enhanced abdominal preoperative staging CT showing no liver nodules.**

The patient was a 59 year old man with a recent history of rectal bleeding and change in bowel habits. The patient had previous medical history of dyslipidemia and was not taking any medication. Family history was unremarkable for neoplasms. Physical examination showed a patient in good general status, and palpation of the abdomen and rectal examination were normal. Colonoscopy showed a stenotic, ulcerated lesion at 20 cm of the anal verge and biopsy revealed a moderately differentiated adenocarcinoma. Laboratory investigations showed normal CEA (1.9 ng/mL) and CA 19.9 (14 U/mL). Liver function tests were normal, apart from slight elevation (130 U/L) of gama-glutamyl transpeptidase [(GGT) normal < 55]. Computed Tomography (CT) of the chest, abdomen and pelvis revealed no distant metastases (Figure 1).

Sigmoidectomy was performed and the postoperative period was complicated by anastomotic breakdown and pelvic sepsis. Re-laparotomy, drainage and loop ileostomy were performed but the postoperative course was otherwise uneventful. Pathology showed a well differentiated adenocarcinoma (T3N1bMx) of the rectum-sigmoid transition and the patient underwent 12 cycles of adjuvant chemotherapy with capecitabine and oxaliplatin.

**Figure 2:**
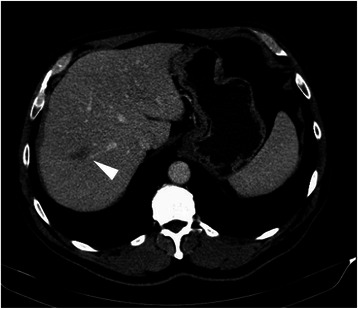
**Contrast enhanced CT after 12 cycles of oxaliplatin-based adjuvant chemotherapy showing a new liver nodule in segment 8, in close proximity to the right hepatic vein (arrowhead).**

One year after surgery, a 28 mm hypodense liver nodule on segment 8 was discovered on routine postoperative CT (Figure 2); this nodule was not present on preoperative CT. Blood levels of CEA and CA 19.9 remained normal (1.6 ng/mL and 11 U/mL respectively). Liver function tests were unremarkable, with a sustained elevation of GGT (143 U/L). A diagnosis of liver metastasis was made and after discussion in our multidisciplinary meeting a resection was proposed (and accepted) to the patient.

During laparotomy, perioperative ultrasound confirmed a 30 mm hyperechoic lesion in segment 8, not typical of metastases, and no other liver lesions. A right hepatectomy and closure of loop ileostomy were performed. Postoperative period was uneventful and the patient was discharged on the 6^th^ postoperative day. No further treatment was prescribed.

**Figure 3:**
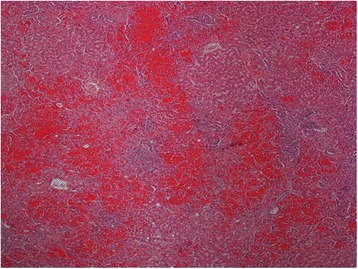
**Panlobular diffuse sinusoidal dilatation and congestion (H&E 40x).**

**Figure 4:**
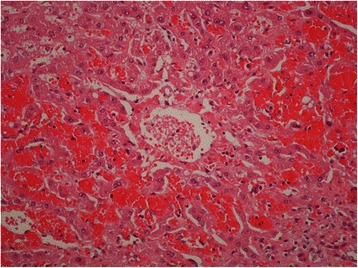
**Normal terminal hepatic veins (H&E 200x).**

**Figure 5:**
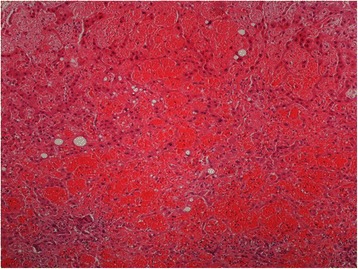
**Extreme sinusoidal dilatation with peliosis (H&E 100x).**

**Figure 6:**
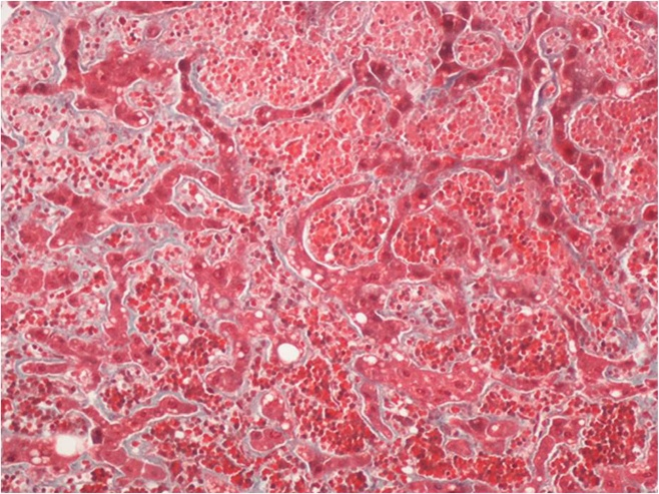
**Atrophic hepatocelular plates and perisinusoidal fibrosis (Trichrome Masson 200x).**

On gross examination the liver parenchyma had congestive areas with no evidence of nodule. Histologically there was haphazardly distributed sinusoidal congestion and dilatation with variable intensity. In some areas sinusoidal dilatation was intense with hemorrhage, trabecular atrophy, focal peliosis and perisinuosoidal fibrosis and the hepatic veins were normal. (Figures 3, 4, 5 and 6).

One year after liver resection and two years after resection of the primary the patient is well, without clinical, biological or radiological evidence of recurrence.

## Discussion

Oxaliplatin based chemotherapy is commonly used in the adjuvant setting in colorectal cancer and as conversion or neoadjuvant therapy in colorectal cancer liver metastases (CRCLM) [1,2]. A well-documented side effect is Sinusoidal Obstruction Syndrome (SOS), formerly known as venoocclusive disease, a recognized complication of bone marrow transplantation; but also of chemotherapy with oxaliplatin [3,7].

Pathophysiology of SOS is still not completely understood but is probably related to the development of a pro-thrombotic state associated with up-regulation of plasminogen activator inhibitor 1 (PAI.1), vonWillenbrand factor (vWF) and Matrix Metalloproteinases (MMP’s) [8-10] The role of angiogenesis is probably confirmed by the presence of sinusoidal capillarization and the protective role of bevacizumab, an antiangiogenic monoclonal antibody [3,11].

SOS is characterised by hepatic sinusoidal dilatation, hepatocyte atrophy, peri-sinusoidal fibrosis, and nodular regenerative hyperplasia. These histological changes appear to be present in up to 40% of patients treated with oxaliplatin based regimens undergoing liver resection; and by causing portal hypertension, they increase the risk of perioperative bleeding and postoperative liver failure [4,11,12].

Although SOS can cause nodular regenerative hyperplasia, the finding of a nodule mimicking liver metastasis has been, to the author’s knowledge, only described in the literature in two precedent publications. In the report by Uchino *et al.* [5], multiple liver nodules were discovered after only six cycles of fluorouracil and oxaliplatin adjuvant chemotherapy. The nodules were all less than 20 mm in size and all in the right liver. Magnetic Resonance Imaging (MRI) showed hyperintense lesions on T2-weighted images. There was however no restriction to diffusion which, in retrospective and in the author’s opinion, would argue against metastases. During operation resection of one of the nodules was performed and frozen section pathological exam showed severe sinusoidal dilation. The remaining lesions were not resected and regressed after stopping chemotherapy.

Xiong *et al.* [6] describe a single nodule detected as hypodense lesion in segment 8, also not present in previous CT. MRI confirmed the lesion as hyperintense on T2 and without enhancement after gadolinium constrast administration. Diffusion-weighted imaging was not reported in this case. The lesion presented metachronously one year after surgery of the primary and after adjuvant chemotherapy with capecitabine and oxaliplatin. There was also no elevation in CEA or CA 19.9 levels. A presumptive diagnosis of CRCLM was made and hepatectomy was performed. Pathology showed peliosis hepatis, a lesion in the spectrum of SOS. However, there is no pathological description of sinusoidal dilation.

In our case the nodule was detected 12 months after surgery of the primary and 6 months after the end of chemotherapy. There was no elevation in tumour markers and the lesion was detected as a hypodense lesion with 28 mm in the right liver. Diffusion-weighted MRI could have been done in order to investigate this nodule, and is nowadays performed more frequently in our centre. An alternate differential diagnosis would be of a necrotic nodule but distinction from a CRCLM is sometimes difficult [13]. Intraoperative ultrasound displayed a hyperechoic mass in segment 8, which is unusual in colorectal liver metastases. Finally, in our patient the central location of the nodule, in segment 8 close the right hepatic vein precluded frozen section examination or minor hepatectomy and left us with no option but performing right hepatectomy.

To our knowledge this is the third reported case of a liver nodule developing after chemotherapy with oxaliplatin. We suggest that in patients treated with oxaliplatin with *de novo* liver nodules, this differential diagnosis should be considered and further imaging evaluation should be pursued. In particular, diffusion-weighted MRI and PET-CT are important imaging techniques in the management of patients with CRC [14]. The finding of atypical radiologic features not suggestive of liver metastases, such as no restriction to diffusion on MRI, should prompt liver biopsy. Further studies however are needed, in particular on the incidence of SOS, peliosis hepatis and nodular regenerative hyperplasia and on the imaging findings associated with it. We believe that further investigation is currently needed on the pathophysiology of SOS [9,10].

In conclusion, we think this case should alert clinicians treating colorectal cancer patients to a rare but potentially severe complication of oxaliplatin; it can be responsible for a misdiagnosis of liver metastases and for overtreatment of the patients. However, we also caution to the fact that most liver nodules detected during follow-up for colorectal cancer are indeed liver metastases and any decision for resection should be taken in high-volume hepatobiliary centres, after thorough discussion in a multidisciplinary setting.

## Consent

The patient willingly gave his informed consent to the publication of this case report.
